# *Escherichia coli *genes that reduce the lethal effects of stress

**DOI:** 10.1186/1471-2180-10-35

**Published:** 2010-02-04

**Authors:** Xiulin Han, Angella Dorsey-Oresto, Muhammad Malik, Jian-Ying Wang, Karl Drlica, Xilin Zhao, Tao Lu

**Affiliations:** 1 Yunnan Institute of Microbiology, Yunnan University, 52 Cui Hu Bei Lu, Kunming, Yunnan 650091, PR China; 2 Public Health Research Institute, New Jersey Medical School, UMDNJ, 225 Warren Street, Newark, NJ 07103, USA

## Abstract

**Background:**

The continuing emergence of antimicrobial resistance requires the development of new compounds and/or enhancers of existing compounds. Genes that protect against the lethal effects of antibiotic stress are potential targets of enhancers. To distinguish such genes from those involved in drug uptake and efflux, a new susceptibility screen is required.

**Results:**

Transposon (Tn5)-mediated mutagenesis was used to create a library of *Escherichia coli *mutants that was screened for hypersensitivity to the lethal action of quinolones and counter-screened to have wild-type bacteriostatic susceptibility. Mutants with this novel "hyperlethal" phenotype were found. The phenotype was transferable to other *E. coli *strains by P1-mediated transduction, and for a subset of the mutants the phenotype was complemented by the corresponding wild-type gene cloned into a plasmid. Thus, the inactivation of these genes was responsible for hyperlethality. Nucleotide sequence analysis identified 14 genes, mostly of unknown function, as potential factors protecting from lethal effects of stress. The 14 mutants were killed more readily than wild-type cells by mitomycin C and hydrogen peroxide; nine were also more readily killed by UV irradiation, and several exhibited increased susceptibility to killing by sodium dodecyl sulfate. No mutant was more readily killed by high temperature.

**Conclusions:**

A new screening strategy identified a diverse set of *E. coli *genes involved in the response to lethal antimicrobial and environmental stress, with some genes being involved in the response to multiple stressors. The gene set, which differed from sets previously identified with bacteriostatic assays, provides an entry point for obtaining small-molecule enhancers that will affect multiple antimicrobial agents.

## Background

The emergence of antimicrobial resistance is severely limiting treatment options for many important infectious diseases [1,2]. Traditionally the problem of antimicrobial resistance has been approached by developing new compounds having increased potency. Unfortunately, development of new compounds is not keeping pace with the emergence of antibiotic-resistant pathogens. Consequently, new strategies are needed to preserve existing agents. One approach is to seek compounds that will enhance the activity of distinct antimicrobial classes by blocking resistance mechanisms. For example, β-lactamase inhibitors extended the utility of β-lactams when delivered as combinations such as Augmentin (amoxicillin-clavulanic acid) [3], and inhibitors of efflux pumps produced synergistic inhibition of growth against tetracycline-resistant *Escherichia coli *when used in combination with doxycycline [4]. The conventional strategy has been to identify genes whose inactivation increases the ability of compounds to block bacterial growth (decreases in minimal inhibitory concentration, MIC) [5]. Since some compounds kill bacteria by processes that are distinct from bacteriostatic action [6,7] and since deficiencies in repair of lethal damage may not affect bacterial growth, the possibility exists that genes involved in bacterial survival are distinct from those that protect from growth inhibition. Finding genes whose products protect from the lethal effects of stress requires screening procedures that differ from those used for bacteriostatic effects.

In the present work, we used the prototype quinolone, nalidixic acid, as a probe for screening genes whose products protect *E. coli *from lethal effects of stress. Nalidixic acid was chosen as the initial screening agent because bacteriostatic and lethal action are distinct events that are sensitive to different drug concentrations (for review see [8]). Mutants of *E. coli*, obtained by Tn5-mediated insertional mutagenesis, were screened for those that had the same bacteriostatic susceptibility to nalidixic acid as the wild-type strain while exhibiting greater sensitivity to the lethal action of the drug. We call this new phenotype hyperlethality. With this phenotype we could eliminate from consideration mutants with altered drug uptake, efflux, and target interactions, since these properties affect bacteriostatic activity. The decreased survival of the mutants was expected in some cases to arise from disruption of genes involved in protecting from lethal stress. The hyperlethal mutants were then examined by measuring the lethal action of several other antimicrobial and environmental stresses. This work defined a novel bactericidal phenotype and identified a diverse set of poorly characterized bacterial stress-response genes as a new source of potential targets for antimicrobial enhancement.

## Methods

### Bacterial strains and growth conditions

Bacterial strains were derivatives of *E. coli *K-12. Strain AB1157 (isolate KD1045) [9] was used to construct the Tn5-insertion library. Strain DM4100 [10] was used to confirm the hyperlethal phenotype following P1-mediated transduction, which was carried out according to a standard procedure [11]. In this method P1 phage lysates were prepared using the insertion mutants as donors, and the lysates were then used to infect strain DM4100 at a multiplicity of infection of 0.2. Transductants were recovered by growth on LB plates containing 25 μg/ml of kanamycin and 0.01 M sodium citrate. Kanamycin-resistant transductants were tested for the hyperlethal phenotype with nalidixic acid. Bacterial cells were grown at 37°C either in LB broth or on LB agar plates [11].

### Antibacterial agents

All chemicals were from Sigma-Aldrich Corp. (St Louis, MO, USA). Stock solutions of nalidixic acid were prepared by dissolving in 0.1 N NaOH to yield a final concentration of 10 mg/ml. Other antibiotics were dissolved in distilled water except for tetracycline and mitomycin C, which were dissolved in 50% and 70% ethanol, respectively. Mitomycin C was freshly prepared before use; other antimicrobials were stored as concentrated stock solutions at -80°C.

### Library construction and screening

Bacteriophage lambda Tn5-tac was prepared from *E. coli *BD1527 [12] according to a standard procedure [13], and Tn5 hopping was carried out with strain AB1157 as follows: recipient cells were grown to mid-log phase (OD_600 _= 0.3 ~0.5), recovered by centrifugation (6,000 × g, 5 min), and resuspended in ice-cold LB liquid medium containing 0.01 M magnesium sulfate. Cells were then infected with lambda Tn5-tac at a multiplicity of infection of about 1 and incubated for 15 min at 37°C. After incubation, fresh LB medium was added, and the cells were incubated for 2 hr at 37°C for expression of kanamycin resistance. Cells were then plated on LB-agar plates containing 25 μg/ml of kanamycin. After incubation overnight at 37°C, kanamycin-resistant colonies were tested individually for nalidixic acid susceptibility (MIC) and lethality as described below. Mutants that were more readily killed by treatment with nalidixic acid at 20 μg/ml or 50 μg/ml for 2 hr but had MICs close to wild-type levels were considered to have a hyperlethal phenotype; they were selected for further analysis.

### Determination of antimicrobial susceptibility and lethality

Antimicrobial susceptibiltiy (MIC_99_) was defined as the minimal concentration of antimicrobial agents that inhibited growth of 99% of the input cells. MIC_99 _was measured by applying 10 μl of serial dilutions of mid-log phase cultures (OD_600 _= 0.3 ~0.5) in triplicate to LB agar plates containing various concentrations of antimicrobials. Colonies were counted after overnight incubation at 37°C. The fraction of colonies recovered (relative to the CFU per ml on drug-free plates) was plotted against drug concentration, and MIC_99 _was determined by interpolation.

To measure lethality, cells were grown in LB liquid medium to mid-log phase (OD_600 _= 0.3 ~0.5) at 37°C with shaking. Cells were split into 1-ml aliquots in test tubes, and various concentrations of antimicrobial agents (2 × MIC_99 _to 30 × MIC_99_) were added. After incubation for 2 hr with shaking, cells were diluted in LB liquid medium, which eliminated drug carryover, and 10 μl of aliquots from the dilutions were spotted in triplicate on drug-free LB agar plates. Colonies were counted after overnight incubation at 37°C. Lethality was expressed as percent of control relative to the CFU per ml at the time of drug addition. The dose that reduced CFU by 90% was taken as LD_90_.

For screening the mutant library, kanamycin-resistant colonies were manually replica-plated with toothpicks to a series of plates containing various concentrations of nalidixic acid and incubated overnight. Colonies exhibiting the same bacteriostatic susceptibility as the parental strain were saved for lethality measurement. Survival for each colony was measured in liquid medium after a 2-hr incubation in nalidixic acid at 20 μg/ml and 50 μg/ml as described in the previous paragraph. Colonies that exhibited decreased survival relative to the parental strain were then retested for MIC and survival as described in the previous paragraphs. Strains confirmed to have a hyperlethal phenotype were further characterized as described below.

### Identification of gene insertion sites

Asymmetric PCR, modified from that described previously [14-16], was used to amplify *E. coli *genomic sequences near the ends of Tn5 that inserted into the genome. One primer, either Tn5R10 (5' GGG ATC CCC TAC TTG TGT AT 3') or Tn5F4568 (5' AGA ATT CCT CCC GAG ATC TG 3') was complementary to the sequence at an end of Tn5; the other primer contained a 6-nucleotide random sequence followed by TGGC (Ran5-29: 5' GTT CTA CAC GAG TCA CTG CAG NNN NNN TGG C 3'). The randomized primer binds any GCCA in the genome. However, since PCR preferentially amplifies short fragments, combination of the two primers should amplify the sequences between one Tn5 end and the first few GCCA sequence elements. For the first 5 cycles of PCR, the annealing temperature was high (58°C); consequently, the primer that was complementary to the sequence at the Tn5 end preferentially bound to the substrate, which caused one strand of the substrate to be asymmetrically amplified. This high-temperature annealing was followed by a cycle using low annealing temperature (30°C) to allow the randomized primer to bind the strand that had already been amplified. Then one high-temperature (58°C) and one moderate-temperature (44°C) cycle were alternated 12 times to amplify the sequence between the two primers. For all amplification cycles, the annealing time was 1 min, while the denaturation (94°C) and extension (72°C) times were 15 sec and 2 min, respectively. The nucleotide sequence was then determined for PCR products that exhibited single bands following agarose gel electrophoresis. In some cases, the products of the first PCR were further amplified with repeated alternation of one high annealing temperature (58°C) cycle and one moderate annealing temperature (44°C) cycle in which the randomized primer was replaced with primer Fix5-29-2 (5' CTA CAC GAG TCA CTG CAG 3'), a primer sequence that was identical to 18 of the 21 5' terminal nucleotides of the randomized primer. DNA sequences obtained were used as query probes to search the *E. coli *K-12 genome sequence database for identifying transposon insertion sites.

### Lethality of environmental stresses

The susceptibility of bacterial cells to UV irradiation was tested by applying serial dilutions of mid-log phase (OD_600 _= 0.3 ~0.5) cultures to agar plates that were irradiated with an Ultraviolet Crosslinker CL-1000 (UVP) at a dose of 2000 μJ/cm^2 ^in a dark room. The plates were then covered with aluminium foil and incubated overnight at 37°C. For other stressors, mid-log phase cells were treated with 2 mM H_2_O_2 _(cells were resuspended in 0.9% saline before treatment), 10% sodium dodecyl sulfate (SDS), or high temperature (52°C) for 15 min. Serial dilutions were then prepared, and 10-μl of aliquots from the dilutions were spotted in triplicate on plates and incubated at 37°C overnight. The sensitivity of cells to the lethal effects of these stressors was expressed as percent survival of treated cells relative to that of untreated cells determined at the time of treatment (LD_90 _could not be used because many of the mutant-stressor combinations did not reduce survival sufficiently).

### Complementation of hyperlethality by cloned genes

All DNA manipulations were carried out according to procedures described previously [13]. The *emrK *and *ycjU *genes with their promoter regions were amplified by PCR using chromosomal DNA isolated from DM4100 as templates and cloned into pBR322. The primers used were 5'-TAG GAA TTC ATC TCC CTT CTC CCT GTA GT-3' and 5'-TAA GTC GAC ATT CTT TGT GCC AAC CTG-3' for *emrK*, and 5'-TGC GAA TTC CTG CTG ACC CAA AGT TAT-3' and 5'-TAG CTG CAG TCA CCT CTT TGG CGA TT-3' for *ycjU*. Plasmids containing wild-type *ycjW*, *yrbB*, and *ybcM *were from the ASKA library [17]. The plasmids were placed in the corresponding mutant strains, as well as in the wild-type strain DM4100, by electroporation. The strains harboring the plasmids were then tested for nalidixic acid lethality. For *ycjW*, *yrbB*, and *ybcM*, the expression was induced by adding 1 mM of IPTG 2 hr before nalidixic acid treatment.

## Results and Discussion

### Screening for mutants exhibiting hyperlethality to nalidixic acid

**Figure 1:**
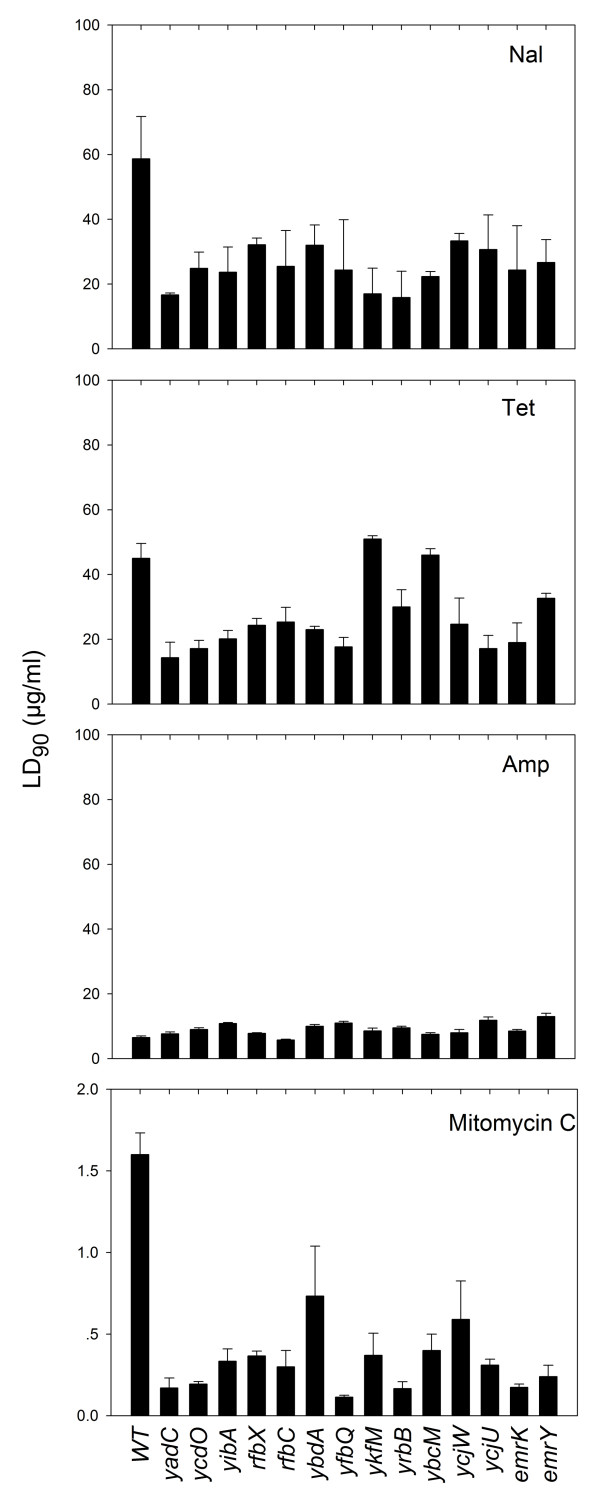
**Antimicrobial susceptibilities of insertion mutants**. *E. coli *cultures grown to mid-log phase were treated with various concentrations of antimicrobial agents for 2 hr at 37°C. Bactericidal activity was expressed as percent survival relative to the CFU per ml at the time of drug addition. The concentration that reduced CFU by 90% was taken as LD_90_. The values are the means of 3 independent experiments. Error bars indicate standard deviations of means.

During the course of evolution, bacteria have acquired a variety of genetic networks that provide protection from stress. For example, in *E. coli *more than 30 two-component systems detect the environment and cause changes in the expression of large numbers of genes [18]. To find genes that are specifically involved in protecting from the lethal action of antibiotics, as opposed to genes that simply limit drug uptake or enhance efflux, we screened a library of transposon-generated mutants for a hyperlethal phenotype (more readily killed by nalidixic acid with no change in bacteriostatic susceptibility, MIC). From about 800 insertion mutants we recovered 14 that exhibited the phenotype. To establish that the hyperlethal phenotype arose from transposon insertion, each of the mutations was transferred to a second strain of *E. coli *by P1-mediated transduction. Transductants from each mutant strain were more readily killed by nalidixic acid (Fig. 1) while displaying less than a 2-fold variation in MIC_99 _relative to the wild-type parent (Table 1). Thus, the Tn5-insertion was necessary and sufficient for the hyperlethal phenotype with all 14 mutants tested.

**Table 1:** Properties of genes that reduce the lethal effects of stress.

Strain	MIC_99 _of Nal (μg/ml)^a^	Site of insertion	Functional annotation of disrupted genes
DM4100	4.5 ± 0.3	NA (wild-type)	NA
TL17	3.1 ± 0.1	*yadC*	Fimbrial-like protein
TL18	4.6 ± 0.3	*ycdO*	Putative lipoprotein
TL19	4.2 ± 0.6	*yibA*	Predicted lyase containing HEAT-repeat
TL20	4.6 ± 0.4	*rfbX*	RfbX lipopolysaccharide PST transporter
TL21	4.8 ± 0.2	*rfbC*	dTDP-4-deoxyrhamnose-3,5-epimerase
TL22	4.7 ± 0.1	*ybdA*	Permease (major facilitator superfamily (MFS) of transporters)
TL23	3.7 ± 0.3	*yfbQ*	Predicted aminotransferase
TL24	3.3 ± 0.2	*ykfM*	Predicted protein
TL25	3.0 ± 0.2	*yrbB*	Predicted NTP-binding protein
TL26	5.3 ± 0.3	*ybcM*	ARAC-type regulatory protein
TL28	3.4 ± 0.1	*ycjW*	Putative LACI-type transcriptional regulator
TL157	4.1 ± 0.5	*ycjU*	Putative β-phosphoglucomutase
TL158	4.0 ± 0.6	*emrK*	Putative membrane fusion protein
TL162	4.4 ± 0.6	*emrY*	Putative multidrug MFS transporter

^a^MIC_99 _was measured by applying serial dilutions of mid-log phase cultures to agar plates containing various concentrations of nalidixic acid followed by incubation, colony number determination, and MIC_99 _estimation as described in Methods. The values shown are the means of 3 independent experiments with standard deviations as indicated. Abbreviations: Nal: nalidixic acid; NA: not applicable.

To identify the genes inactivated by Tn5 insertion, asymmetric PCR was used to amplify the sequences near the ends of Tn5 using a protocol modified from previously published reports [14-16]. Nucleotide sequence determination of the PCR products then identified 14 different genes (Table 1). Only one of these genes (*ycjU*, also called *pgmB*) appeared in the list of genes identified previously as being involved in affecting the bacteriostatic action of quinolones [5]. The *ycjU *mutation caused cells to be only slightly more susceptible to nalidixic acid than the wild-type strain in our bacteriostatic measurement (Table 1, MIC_99 _4.1 μg/ml vs. 4.5 μg/ml). Thus, *ycjU *may not belong in the set previously identified as having a low MIC [5]. The two-fold drop in LD_90_, from 59 μg/ml to 31 μg/ml (Fig. 1), qualified it as a gene with a hyperlethal phenotype.

### Mutant susceptibility to other antimicrobial agents and environmental stressors

To determine whether the hyperlethal phenotype was restricted to quinolones, we examined the response of the mutants to a variety of other stresses. When tetracycline was tested, we found that, except for two strains TL24 (*ykfM*::Tn5) and TL26 (*ybcM*::Tn5), the mutants were more readily killed (LD_90 _was about half the wild-type value, Fig. 1). Increased lethality was not observed for the β-lactam ampicillin (Fig. 1). Thus, increased killing of the mutants by antimicrobial agents was not restricted to quinolones, but it was also not universal.

**Figure 2:**
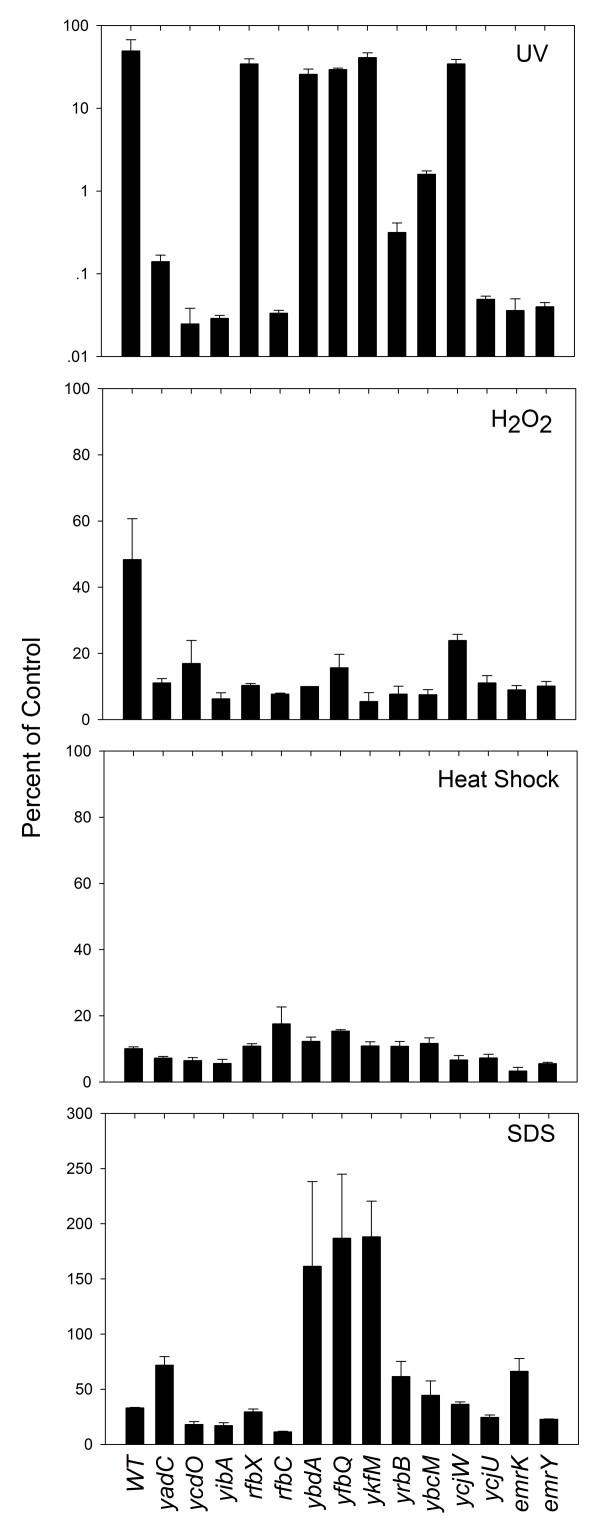
**Susceptibilities of insertion mutants to physical and chemical stresses**. *E. coli *cultures grown to mid-log phase were treated with 2000 μJ/cm^2 ^of UV; 2 mM H_2_O_2_, 10% SDS, or heat shock at 52°C for 15 min. Samples were diluted, applied to agar lacking stressor, and incubated to determine the fraction of colonies surviving. This fraction was expressed as a percent of an untreated control culture sampled at the time stress was applied. In the case of SDS, some mutants grew during treatment, which caused those samples to have values higher than the control. Values reported are the means of 3 independent experiments. Error bars indicate standard deviations of means.

When the DNA damaging agent mitomycin C was tested, all of the mutants were more readily killed than wild-type cells (for some genes LD_90 _was 10% of wild-type values, many were in the 20 to 30% range, and two were close to 50%, Fig. 1). More than half of the mutants were more readily killed by UV irradiation than the wild-type strain (Fig. 2). Genes not involved in protecting cells from the effects of UV irradiation were *rfbX*, *ybdA*, *yfbQ*, *ykfM*, and *ycjW*. Nine others were involved in protecting cells from the effects of nalidixic acid, mitomycin C, and UV. Thus, many of the genes are involved in facilitating survival of *E. coli *cells exposed to DNA-damaging agents.

The effect of hydrogen peroxide was also examined, since it has recently been implicated in the lethal action of multiple antibiotics [6,7]. After treatment with 2 mM H_2_O_2 _for 15 min, all mutants showed decreased survival compared to wild-type strain DM4100 (for many mutants survival was 20 to 30% that of the wild-type strain, Fig. 2). We also examined the effects of two other environmental stresses, exposure to high temperature and to the ionic detergent sodium dodecyl sulfate (SDS). When the set of mutants was exposed to high temperature (52°C), no substantial difference among the mutants was observed for heat-induced death (Fig. 2). With 10% SDS, the effect of the mutations on survival varied. Some mutants (*yadC*, *ybdA*, *yfbQ*, *ykfM*, *yrbB*, *ybcM*, and *emrK*) were less susceptible to killing than wild-type *E. coli*, while *ycdO, yibA*, and *rfbC *mutants were more readily killed (Fig. 2).

In summary, 14 mutant genes were associated with hyperlethality to nalidixic acid and were more readily killed by mitomycin C and peroxide; 9 were more readily killed by UV irradiation. Only 3 of the mutants were more readily killed by SDS, none by high temperature. Below we consider what has been reported previously about the diverse set of genes identified by our screening procedure. None of that information leads to an expectation of hyperlethality to nalidixic acid.

### Putative function of genes exhibiting hyperlethality to nalidixic acid

Eight of the mutant genes (*yadC, rfbX*, *rfbC*, *ycdO, yrbB*, *ybdA*, *emrK*, and *emrY*) were annotated in Genbank as outer membrane proteins or proteins whose function is related to the outer membrane. The MIC_99_s of these mutants for nalidixic acid were in the same range as that of the wild-type strain; consequently, hyperlethality caused by these mutations was unlikely to be due to increased accumulation of drug. Since the genes were involved in protecting from the effects of nalidixic acid, mitomycin C, and hydrogen peroxide, it is likely that protection from UV irradiation also occurred at the level of downstream effects of irradiation rather than through screening cells from UV light.

The *yadC *mutant, which was among the more sensitive to DNA damaging agents, was considerably less sensitive than the wild-type strain to SDS. YadC is a fimbrial-like protein whose amino acid sequence suggests that it may contain β-barrel structure(s) [19]. In *E. coli*, pili are adherence factors that could, in principle, protect some cells in a population from antimicrobial treatment. However, we detected no difference with respect to cellular aggregation when the *yadC *mutant and the wild-type strain, growing exponentially, were examined by light microscopy (not shown). Thus, the hyperlethal phenotype of the mutant was not likely to be due to lack of cellular self-association. *yadC *is induced immediately after exposure to the biocide polyhexamethylene biguanide [20], which is consistent with its involvement in a cellular response to stress.

The two *rfb *mutants were strikingly different in their response to UV: the *rfbX *mutant showed little effect, while the *rfbC *mutant was one of the most susceptible (Fig. 2). RfbX, also known as WzxB, is a member of the polysaccharide transporter (PST) family [21], and hydropathy analysis suggests that RfbX has 12 transmembrane segments [22]. *rfbC *encodes dTDP-4-dehydrorhamnose 3,5-epimerase [23]. Although the *rfb *genes are thought to be involved in O-antigen biosynthesis in enteric bacteria, such as *Salmonella*, *Shigella*, *Klebsiella*, and some serovars of *E. coli *[24], that feature may be irrelevant to the current work, since *E. coli *K-12 does not normally express a functional O-antigen due to defects in the *rfb *gene cluster [25,26]. The fact that *rfbX *is induced when treated with polyhexamethylene biguanide [20] suggests that it may be involved in a cellular stress response.

The *ycdO *mutant was the most readily killed by UV irradiation (Fig. 2). YcdO is a monomeric, membrane-associated protein that contains a type I signal peptide and is transported to the periplasm with high efficiency [27]. Since YcdO is induced at low pH due to phosphorylation of the CpxR component of the CpxAR two-component response regulator [28-30], it may be involved in bacterial envelope stress responses mediated by the CpxAR pathway.

YrbB(MlaB) is predicted to be a nucleotide-binding protein that contains a STAS domain [31]. YrbB is a component of an ABC transport system that maintains lipid asymmetry in the Gram-negative outer membrane by preventing surface exposure of phospholipids [32]. Although the YrbB mutant is more sensitive than the wild-type strain to the presence of EDTA-SDS, it does not show hypersusceptibility to erythromycin, rifampicin, bacitracin, or novobiocin [32]. These data indicate that the *yrbB *mutation does not change the permeablility to most compounds, which is consistent with our finding that the *yrbB *mutation did not change the bacteriostatic effects of nalidixic acid.

*ybdA*, *emrK*, and *emrY *are postulated to encode efflux pumps, although MIC determinations with nalidixic acid showed no evidence that the mutations affected efflux (Table 1). The YbdA protein is a member of the major facilitator superfamily (MFS) of transporters [33], while EmrK and EmrY show sequence similarity to members of the EmrAB-TolC drug efflux system. The two *emr *mutants were exceptionally sensitive to mitomycin C (Fig. 1), UV irradiation, and H_2_O_2 _(Fig. 2). The *ybdA *and *emrK *mutants were among the least susceptible to the lethal effects of SDS (Fig. 2), as expected of efflux mutants. Perhaps the ErmK, ErmY, and YbdA proteins normally pump out toxic metabolites induced by DNA damage.

Two genes,*ybcM *and *ycjW*, are predicted to be involved in cell regulation. YbcM is a putative DNA-binding transcriptional regulator that is induced by the biocide polyhexamethylene biguanide [20]; in *Yersinia enterocolitica *a homolog of *ybcM *is induced by low temperature [34]. These properties are consistent with YbcM being involved in bacterial stress responses. YcjW is a putative LacI-type transcriptional regulator. Although the biological role of YcjW is unknown, *ycjW *transcripts accumulate in a strain harboring wild-type *relA *but not in a *relA *mutant after treatment with 4-azaleucine, an inhibitor that interferes with translation [35]. These data suggest that YcjW may be involved in the bacterial stringent response.

Three other genes, *ycjU*, *yibA*, and *yfbQ*, encode putative cytosolic proteins of unknown function. The *ycjU *mutant is among the most sensitive to UV irradiation (Fig. 2). YcjU has been annotated in sequence data bases as a putative β-phosphoglucomutase that belongs to the superfamily of haloacid dehalogenase (HAD)-like hydrolases. *In vitro*, YcjU hydrolyzes small phosphodonors [36], which suggest that the protein is likely to have other physiological roles. The *yibA *mutant was among the most sensitive to UV irradiation and H_2_O_2 _(Fig. 2). YibA is a predicted lyase containing a HEAT-repeat, which forms a rod-like helical structure in proteins. Transcription profiling experiments suggested that *yibA *may belong to the σ^32 ^regulon [37], whose genes are expressed in *E. coli *in response to heat shock. Thus, the role of YibA in antimicrobial susceptibility may be exerted through alternative sigma factor-regulated stress responses. However, the *yibA *mutant was not particularly sensitive to high temperature. A third mutant, in *yfbQ*, was the most sensitive to mitomycin C. The only information available refers to the gene product as a potential aminotransferase.

### Reactive oxygen species-mediated response to lethal antimicrobials

Although no clear metabolic connection exists among the genes we identified, some guidance can be gained from the recent proposal that lethal antimicrobials share a common cell death pathway involving a reactive oxygen cascade [6,7]. The lethal activity of a variety of antimicrobials, including the fluoroquinolone norfloxacin, is accompanied by an increase in hydroxyl radical, and lethal activity is greatly reduced by treating *E. coli *cells with agents that block the accumulation of hydroxyl radical [6]. The idea emerged that lethal antimicrobials act in part by generating a signal that causes an accumulation of superoxide, which reacts with iron-sulfur clusters to release peroxide and iron. Peroxide and iron then form highly toxic hydroxyl radicals through the Fenton reaction. Superoxide can also be converted to peroxide by superoxide dismutase and by spontaneous dismutation. The resulting increase in peroxide would contribute to the formation of hydroxyl radical. In support of this idea, we found that deletion of both superoxide dismutase genes reduced the lethality of norfloxacin [38]. As expected, a deficiency of catalase, which converts peroxide to water, led to an increase in the lethality of norfloxacin [38]. Mutations in genes that normally protect from the accumulation of reactive oxygen species would be recovered by our screen for hyperlethality to nalidixic acid. Such mutants are expected to also be more readily killed by other DNA damaging agents, such as mitomycin C, peroxide, and UV irradiation, as seen for 9 of the 14 of the genes we identified.

### Complementation of hyperlethality by cloned genes

**Figure 3:**
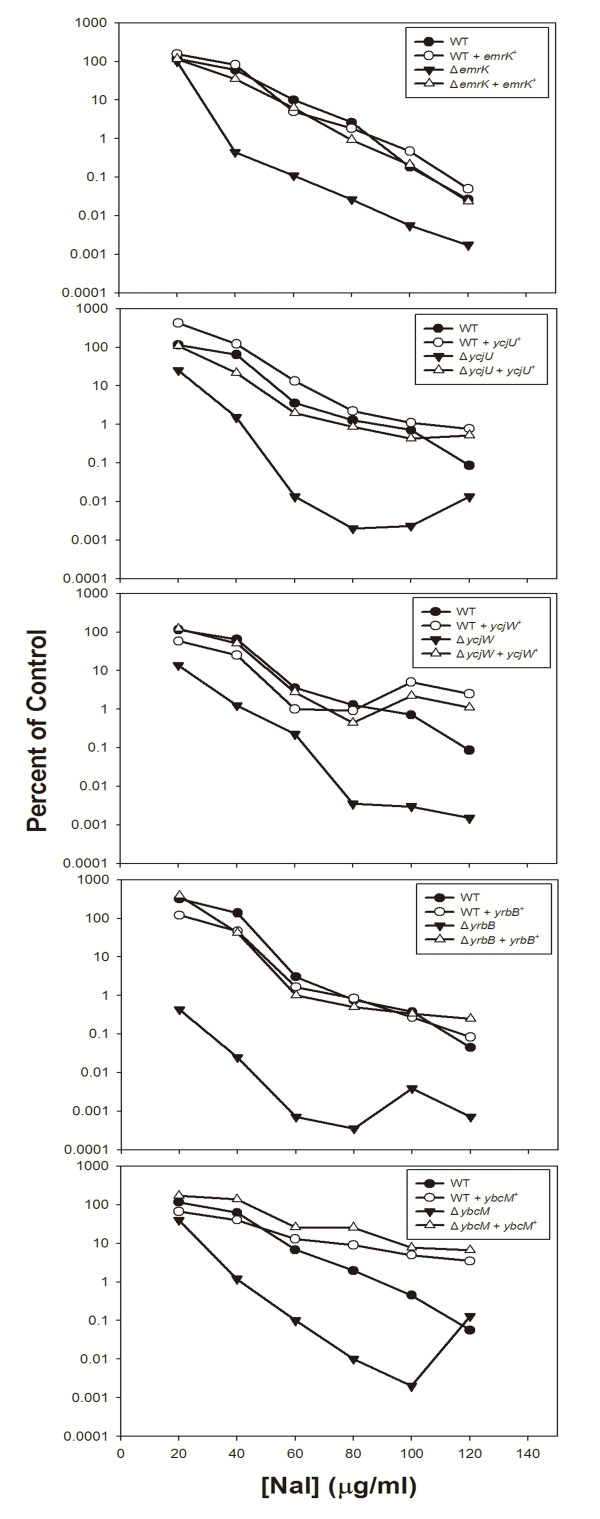
**Complementation of hyperlethal phenotype by cloned genes**. Plasmids containing wild-type genes were transformed into the corresponding Tn5-containing mutants. The strains harboring the plasmids were then tested for nalidixic acid-mediated lethality by treating mid-log phase cells with various concentrations of nalidixic acid for 2 hr at 37°C. Percent of control indicates percent survival of treated cells relative to untreated cells sampled at the time of drug addition. For *ycjW*, *yrbB*, and *ybcM*, the expression was induced by adding 1 mM of IPTG 2 hr before nalidixic acid treatment. Similar results were obtained in a replicate experiment.

To determine whether the hyperlethal phenotype of the mutants was caused by deficiency of the mutant genes rather than polar effects due to Tn5 insertion, we selected several mutants for complementation using wild-type genes cloned into plasmids. We focused on representatives from three groups (membrane and related proteins (*emrK*, *yrbB*), cytosolic enzymes (*ycjU*), and regulators (*ycjW*, *ybcM*)). In each case, complementation was observed (Fig. 3). Thus, at least for this selection of genes it is likely that the gene products contributed to reducing the lethal effects of nalidixic acid. While these data do not assure that complementation will occur in the other cases, they give us confidence to move forward with the study of the bacterial response to lethal stress. We note in some cases paradoxical survival occurred at high concentrations of nalidixic acid. This phenomenon, which is unexplained, is commonly observed with quinolones [39].

## Conclusions

The present work described a novel screening process for identifying genes involved in protecting *E. coli *from quinolone-mediated death due to events occurring after formation of quinolone-gyrase-DNA complexes. Using this screen we identified 14 poorly characterized genes. Scattered evidence suggests that many of these genes are linked to protective stress responses, which is supported by our finding that mutations in these putative protective genes resulted in decreased survival following treatment with several stressors. The diverse set of genes described may serve as potential targets for future screening of small-molecule antimicrobial potentiators.

## Authors' contributions

XH screened for hypersusceptible mutants, helped identifying insertion sites, and measured susceptibility of mutants to antimicrobial agents and other stresses. AD participated in writing the manuscript. MM participated in mutant screening. JW identified genes containing Tn5 insertions. KD participated in initial project design, supervised all work performed at PHRI, and participated in writing the manuscript. XZ participated in project design, screened for mutants, and participated in writing the manuscript. TL participated in initial project design, supervised all work performed at YNU, constructed the insertion library, screened for mutants, carried out P1-transduction, and carried out primary writing of manuscript. All authors read and approved the final manuscript.
